# Predictors of Workplace Bullying and Cyber-Bullying in New Zealand

**DOI:** 10.3390/ijerph13050448

**Published:** 2016-04-27

**Authors:** Dianne Gardner, Michael O’Driscoll, Helena D. Cooper-Thomas, Maree Roche, Tim Bentley, Bevan Catley, Stephen T. T. Teo, Linda Trenberth

**Affiliations:** 1 School of Psychology, Massey University, Palmerston North 4442, New Zealand; 2 School of Psychology, University of Waikato, Hamilton 3240, New Zealand; m.odriscoll@waikato.ac.nz (M.O.); mroche@waikato.ac.nz (M.R.); 3 School of Psychology, University of Auckland, Auckland 1142, New Zealand; h.cooper-thomas@auckland.ac.nz; 4 School of Management, Massey University, Palmerston North 0745, New Zealand; T.A.Bentley@massey.ac.nz (T.B.); b.e.catley@massey.ac.nz (B.C.); 5 School of Management, RMIT University, Melbourne 3001, Australia; Stephen.teo2@rmit.edu.au; 6 Griffith Business School, Griffith University, Queensland 4111, Australia; l.trenberth@griffith.edu.au

**Keywords:** conservation of resources, bullying, cyber-bullying

## Abstract

*Background*: The negative effects of in-person workplace bullying (WB) are well established. Less is known about cyber-bullying (CB), in which negative behaviours are mediated by technology. Drawing on the conservation of resources theory, the current research examined how individual and organisational factors were related to WB and CB at two time points three months apart. *Methods*: Data were collected by means of an online self-report survey. Eight hundred and twenty-six respondents (58% female, 42% male) provided data at both time points. *Results*: One hundred and twenty-three (15%) of participants had been bullied and 23 (2.8%) of participants had been cyber-bullied within the last six months. Women reported more WB, but not more CB, than men. Worse physical health, higher strain, more destructive leadership, more team conflict and less effective organisational strategies were associated with more WB. Managerial employees experienced more CB than non-managerial employees. Poor physical health, less organisational support and less effective organisational strategies were associated with more CB. *Conclusion*: Rates of CB were lower than those of WB, and very few participants reported experiencing CB without also experiencing WB. Both forms of bullying were associated with poorer work environments, indicating that, where bullying is occurring, the focus should be on organisational systems and processes.

## 1. Introduction

Workplace bullying is known to have severe negative effects on targets, witnesses and organisations. Targets and witnesses can experience stress, anxiety and poor physical and mental health [1,2,3,4], and these effects persist over time [5]. Targets risk being excluded from working life because of ill health, stigmatisation, victimisation or reputational damage [6]. Organisations experience poor employee productivity, as perpetrators are using employer resources and spending time on non-work activities [7], and targets and witnesses are likely to work less efficiently, take more time off, feel less committed to the organisation and be more likely to leave [2,8,9]. Despite the severe problems caused by workplace bullying, organisations have difficulty dealing with it. While policies, rules and guidelines are required, they are rarely sufficient: good role models, effective human resources systems [10], committed leadership [11] and a good workplace culture [12] are also essential to combat this complex psychosocial problem.

In the present study, bullying and cyber-bullying were defined as a situation where a person feels they have repeatedly been on the receiving end of negative actions from one or more other people, when it is difficult to defend themselves against these actions [13]. These negative actions could be physical (e.g., shoving) or non-physical (e.g., verbal abuse). A one-off incident is not defined as bullying (adapted from Lutgen-Sandvik *et al.* [13]). Note that this definition includes both ‘traditional’ face to face bullying (here called workplace bullying (WB)) and negative actions carried out online, known as cyber-bullying (CB).

WB is frequently associated with stressful and poorly-organised working conditions. Job insecurity [14], job stressors such as high workloads, role conflict and role ambiguity [15,16], organisational change [17] and lack of autonomy at work can create stress, uncertainty and opportunities for bullying [18,19,20]. Organisations may also condone and support bullying behaviour if the workplace culture over-emphasizes productivity, performance and competitiveness over wellbeing [17,21,22]. While extensive research has identified factors associated with WB [23], much less is known about the factors associated with CB.

The use of electronic media both within the workplace and outside is growing rapidly, associated with increased interest in work and non-work uses of technology. Building on the extensive work into WB, there is growing interest in bullying behaviour, which is mediated by technology. Cyber-bullying (CB) is defined as “inappropriate, unwanted social exchange behaviours initiated by a perpetrator via online or wireless communication technology and devices” [24]. Forms of CB include “anonymous, fraudulent, aggressive, unwanted messages, spreading rumours, hacking into email accounts, threats, harassment, attacks, unwanted phone calls, malicious, abusive messages” [25]. CB has important elements in common with WB. It is repeated and hurtful, it can be intense; it involves an imbalance of power; and it creates feelings of powerlessness [26]. The key difference between them, from this perspective, is that technology is used for CB. However, it has been argued that CB is distinct from, and potentially more harmful than, other forms of bullying, due to three features that WB does not share: potential for anonymity, publicness and access [26].

The potential anonymity of online interactions affects both perpetrators and targets. Cyber-bullies may feel protected and able to act with fewer constraints and, being remote from their targets, may not recognise or may be more able to ignore a target’s distress [3,27,28,29,30,31]. Targets may not know who is bullying them, leading to feelings of helplessness and invasion of privacy [25], fear [32] and being pursued, “haunted” and “hemmed in” [33], with few opportunities for redress [3]. At the other extreme, cyber-bullying can also be extremely public. A cyber-bully can publish comments, allegations, images and other material to an enormous audience almost instantaneously [3,31]. Audiences can include the target’s family, friends, neighbours and colleagues [30], and reputational harm can be done before a target becomes aware of it [30]. Access to targets is also far broader for the cyber-bully than the traditional bully. Cyber-bullies can potentially reach their targets at any time, in any location and by a wide range of means—email, phone, social networking sites and text messaging—making it difficult for targets to avoid bullies without giving up the technologies which, for many, are essential for work and family communication [31].

Like traditional bullying, cyber-bullying is related to higher stress, less optimism, worse job performance and more dissatisfaction [26,31,33,34,35]. Similar processes are likely to be involved as in traditional bullying: imbalance of power, the stigma associated with being targeted, unwillingness to report, fear of being labelled a “victim” [25,36]. In view of cyber-bullying’s anonymity, publicness and access, it has been proposed to have more severe impacts than traditional bullying, but there is little evidence to address this issue. The prevalence of CB is also relatively unknown. While the anonymity and relative immunity of cyber-bullies could facilitate CB, the prevalence of WB has so far been shown to be higher than CB [27]. Farley [34] found that 46.2% of a sample of trainee doctors had experienced at least one act of CB, while Perreault [37], in a survey of adult Internet users, found that 7% self-identified as having experienced CB. Privitera and Campbell [38] found that 34% of their sample of male union members had been bullied face-to-face, and 10.7% had been cyberbullied; all who experienced CB had also experienced face-to-face WB. There are indications that CB is less prevalent than WB and that it may not be creating ‘new’ targets, but instead is most likely to appear in conjunction with WB [32,39], providing an alternative means of attack, but these issues have yet to be resolved.

### The Role of Resources in Preventing Bullying

The conservation of resources model [40] is useful in explaining the impacts of bullying on wellbeing. Resources are “personal characteristics, conditions or energies that are valued by the individual or that serve as a means for attainment of these objects, personal characteristics, conditions or energies” [41]. Resource drain and the inability to replenish resources can lead to stress, poor wellbeing and burnout [42]. Bullying can deplete targets’ resources as they use their time and energy to deal with the problem, which is likely to detract from their focus on work [5]. Resources that can be eroded by exposure to WB include social support, self-efficacy and optimism [43,44]. Loss of resources can lead to a spiral of future losses, inability to regain resources and decreasing wellbeing as employees become increasingly unable to regain resources, find themselves committing more and more resources to dealing with their situation [45]. While bullying can lead to a loss of resources, there is emerging evidence for a reciprocal process: low resources can be related to more bullying when, for example, poor health and high levels of strain make an employee an easier target for bullies [5]. While the body of longitudinal research into the effects of bullying on resources and *vice versa* is growing, it remains small, and the research exploring these issues in the context of cyber-bullying is even smaller. The aims of the present research were therefore to explore the relationship of personal and organisational factors with WB and CB across two time points. As the perpetrators of WB are frequently of higher organisational status than targets, reflecting the role of power in facilitating bullying [46], role (managerial/non-managerial) was included as a control variable in the analyses. The evidence for gender differences in bullying and cyber-bullying is mixed and few firm conclusions can be drawn; gender was included as a control variable in the analyses [35,47].

While bullying can give rise to a loss of resources, there is emerging evidence of a reciprocal process in which bullying gives rise to distress, which gives rise to further bullying [5,48,49]. Resource loss can lead to poor physical and mental health, which in turn reduce the ability to deal with work and other demands [44]. Employees who are performing poorly or are frequently absent may be bullied as a form of social control in response to organisational norm violation [50]. The outcome is an ongoing “loss spiral” of stress, illness, poor performance and absenteeism, leading to more bullying and further reducing targets’ opportunities to regain resources.

Hypothesis 1a: Self-reported job performance at Time 1 will be negatively related to (i) WB and (ii) CB at Time 2.

Hypothesis 1b: Physical health at Time 1 will be negatively related to (i) WB and (ii) CB at Time 2.

Hypothesis 1c: Strain at Time 1 will be positively related to (i) WB and (ii) CB at Time 2.

Hypothesis 1d: Hypothesis 1d: Self-reported absenteeism at Time 1 will be positively related to (i) WB and (ii) CB at Time 2.

As well as personal resources, there is substantial evidence that organisational factors act as resources that predict the occurrence of WB [15,51,52,53,54]. Poor leadership is strongly related to higher rates of bullying [55,56]. Destructive leadership undermines and/or sabotages the organization’s goals, tasks and resources, as well as the effectiveness and/or motivation, wellbeing or job satisfaction of employees [57]. Destructive leaders can be autocratic, ineffective, unethical, incompetent, inconsistent or overly political [58]. In contrast, fair and supportive leadership is associated with less bullying [16]. Ethical leadership is the “demonstration of normatively appropriate conduct through personal actions and interpersonal relationships” [59]. Ethical leaders display appropriate personal and interpersonal behaviours, are good role models and provide a fair and just work environment [59]. Such leaders are likely to foster supportive workplace climates and to prevent or effectively address conflict and bullying [56].

Hypothesis 2a: Ethical leadership at Time 1 will be negatively related to (i) WB and (ii) CB at Time 2.

Hypothesis 2b: Destructive leadership at Time 1 will be positively related to (i) WB and (ii) CB at Time 2.

Perceived organisational support (POS) is the perception by employees that the organization values their contribution and cares about their wellbeing [60,61]. POS is an organisational resource for employees both because it is valued and because it suggests that other resources will be forthcoming as needed. POS can reduce the likelihood of negative behaviours at work [53], reduce the effects of bullying on targets’ intentions to leave [9] and reduce the effects of negative behaviours on wellbeing and health [62]. Bullying and organisational support can co-exist, but rarely come from the same source. Targets may therefore be able to access some social resources to help address bullying [9].

Hypothesis 3: POS at Time 1 will be negatively related to (i) WB and (ii) CB at Time 2.

Organisations require systems and strategies to manage WB and, increasingly, CB. Employer responses to reports of bullying have consistently been found to be inadequate and may range from helping the target, to doing nothing, to retaliating against the person reporting the bullying [63,64]. While organisations need to provide resources to address bullying, even potentially effective systems are of little value if employees are unaware of them, do not use them or do not trust them [65]. Employee perceptions that organisational anti-bullying strategies are effective are expected to be related to less WB and CB.

Hypothesis 4: Perceived effectiveness of organisational strategies at Time 1 will be negatively related to (i) WB and (ii) CB at Time 2.

A positive team climate can provide resources, such as instrumental and emotional support, positive appraisal and feelings of belonging, which facilitate team performance and satisfaction [66]. In contrast, team conflict can arouse strong emotions and give rise to bullying behaviours aimed at achieving goals, emotional release or retaliation [67,68]. Members of a team experiencing conflict will have fewer resources to deal with bullying arising within or outside the team [69].

Hypothesis 5: Team conflict at Time 1 will be positively related to (i) WB and (ii) CB at Time 2.

## 2. Method

Data were collected by means of online surveys using Qualtrics, a secure hosting site. To participate, individuals needed to be currently employed within New Zealand. Data on all variables were collected at two time points 3 months apart. In order to test the current hypotheses, predictor variables at Time 1 and criterion variables at Time 2 were the focus of analysis. Three months was selected as a timeframe suitable for exploring cross-time effects [70] and to minimize common method variance by separating the measurement of the predictor and criterion variables [71]. Ethical approval was obtained via a Low Risk Notification to the Massey University Human Ethics Committee (Approval Date 9 July 2014). .

### 2.1. Participants

All participants resided and worked in New Zealand at both Time 1 and Time 2. Time 1 participants comprised 991 men (40.9%) and 1421 women (58.6%); 12 (5%) did not provide this information). At Time 2, there were 349 men (42%) and 477 women (58%). The mean age at Time 2 was 50 years, and mean tenure was 6.5 years. Seventy-nine percent (1903) self-identified as New Zealand European 1903, 166 (6.8%) as other European, 167 (6.9%) as Maori or Cook Island Maori, 41 (1.7%) as Pasifika, 53 (2.2%) as Chinese, 53 (2.2%) as Indian and 138 (5.7%) identified with other ethnicities. In terms of job role, 293 (12.1%) were senior managers, 348 (14.4%) mid-level managers, 219 (9.0%) first-line supervisors and 1447 (59.7%) were non-managerial employees, and 117 (4.8%) did not provide this information. Role (coded 0 = non-managerial; 1 = managerial) and gender (0 = male, 1 = female) were entered as control variables in the regressions, to test the hypotheses outlined above. Evidence for the relationships between demographic variables and both WB and CB is inconsistent. Younger employees may be more likely to experience CB than older employees due to their more extensive involvement in online activities [72], but there is little evidence for this in the workplace. The relationships between age, WB and CB were explored. In addition, descriptive data on the perpetrators of WB and CB were examined.

### 2.2. Measures

*Workplace bullying* (WB) was measured using the 22-item Negative Acts Questionnaire-Revised (NAQ-R) [73]. This provides a list of 22 negative behaviours (e.g., “someone withholding information which affects your performance”), which are formulated in behavioural terms with no reference to the term bullying [74]. Respondents indicate how often they have experienced each behaviour over the previous 6 months (never = 0 to daily = 4). The mean score across negative acts was computed for each person (α = 0.93). In line with Leymann’s [75] criterion for identifying targets of WB, respondents were classified as “bullied” if they had experienced at least two of the negative behaviours weekly or more often over the past 6 months. Respondents who did not meet this criterion were classified as non-bullied.

*Cyber-bullying* (CB): To date there are few widely-used and well-validated measures of cyber-bullying that are suitable for use in the workplace, and many of those in use are based on the NAQ-R [34,38]. However, the NAQ-R does not include some behaviours that appear particularly relevant to CB (e.g., hacking of personal communications) while others (e.g., being given an excessive workload) do not seem distinctive to CB. Accordingly, we developed our own set of items, after reviewing a range of other measures. CB was measured using a list of 20 negative acts drawn from a range of sources (e.g., “I have received rude, insulting or offensive online communications by people at work”, 1 = never, 5 = daily).

Established criteria for classifying participants as cyber-bullied have yet to emerge. To enable comparisons with WB, the mean score of negative cyber-acts was computed (α = 0.82), then the criterion of having experienced at least two behaviours at least weekly for the last 6 months was applied. For both sets of questions, the negative acts questions were presented without asking participants about their perceptions of themselves as targets of either WB or CB.

*Self-identified bullying* was examined by providing participants with a definition of bullying (given above) and asking whether they considered themselves to have been bullied over the last 6 months (0 = “no” to 4 = “almost daily”). Those who gave any answer other than “no” were then asked who was responsible for the bullying.

To measure *psychological strain*, the 12-item version of the General Health Questionnaire (GHQ-12) [76,77] was used. Respondents indicated how often (0 = not at all to 3 = much more than usual) they had experienced each of 12 psychosocial symptoms in the previous 6 months, e.g., “felt constantly under strain”. Items were coded so that a higher score indicated more strain, and the mean score was calculated (α = 0.86).

*Self-reported job performance* was measured using a single item from Kessler *et al.* [78], “how would you rate … your own overall job performance on the days you have worked during the past 6 months?” (1 = “the worst performance anyone could have at your job”, 10 = “the performance of a top worker”).

*Physical health* was measured by 13 items adapted from Spector [79], e.g., “Over the past 6 months, how often have you experienced… An upset stomach or nausea” (Less than once a month or never = 1; several times per day = 5). Higher scores reflected poorer physical health (α = 0.82).

*Absenteeism*: The single-item measure used by Bentley *et al*. [80] asked approximately how many days over the previous 6 months respondents had been absent unofficially from work (0 = no days, 3 = 6–10 days).

*Ethical leadership* was measured with 10 items (e.g., “my boss listens to what employees have to say”, 1 = never to 5 = always; α = 0.89) [59].

*Destructive leadership* was measured with 20 items, e.g., “my boss has his/her head in the sand”, 1 = never to 5 = always; α = 0.95) [58].

*Perceived organisational support* (POS) was measured with 7 items from Djurkovic *et al.* [9], e.g., “my organisation strongly considers my goals and values” (1 = strongly disagree, 7 = strongly agree; α = 0.96).

The measure of *team conflict* used 8 items adapted from the intragroup conflict scale developed by Jehn [81] (e.g., “there is friction among members in my work unit”, 1 = never, 5 = always; α = 0.95).

To assess the *perceived effectiveness of organisational strategies*, participants were asked to think about what, if anything, their organisation had done to address bullying, whether face to face or online. Twenty items were derived from various sources and collated for this study (e.g., “a policy or procedure that defines workplace bullying and states that it is unacceptable”). For each item participants answered yes, no, don’t know or prefer not to answer, and for items endorsed “yes” they were then asked to rate the effectiveness of that item (1 = very ineffective, 4 = very effective).

### 2.3. Data Analysis

The WB and CB variables were non-normally distributed, with a positive skew of 2.32 (WB) and 8.45 (CB) and kurtosis of 6.81 (SE = 0.170) for WB and 105.94 (SE = 0.171) for CB. Log transformation produced a WB variable of reasonable symmetry (skew = 1.38; kurtosis = 1.63 (SE = 0.170), but the CB variable remained non-normally distributed (skew = 4.24; kurtosis 27.52; SE = 0.171). Regression and correlational analysis were run with transformed and non-transformed variables; no differences were observed in the patterns of findings, and so, the analyses using non-transformed variables are reported. Results of Harman’s single-factor test did not show a single factor accounting for the majority of covariance among the variables; in addition, predictor and criterion variables were separated in time, which can help to minimize common method variance.

## 3. Results

We examined whether participants who only took part in the first wave of data collection (*n* = 1589) differed in regard to demographic variables from those who completed both waves (*n* = 826; Table 1). The samples did not differ in regard to their demographic characteristics, except that fewer senior managers/executives completed the survey at both time points than at Time 1 alone (16.2% *vs.* 6.4%), while more non-managerial employees did so (59.7% *vs.* 62.7%; chi-square (3) = 46.7, *p* < 0.001).

Using the criterion of having experienced two or more negative acts at least weekly for at least six months, 123 (15%) of participants had been bullied and 23 (2.8%) of participants had been cyber-bullied. Six hundred and eighty-eight participants (84%) had experienced neither WB nor CB according to these criteria, whereas 20 (2%) had experienced both; 100 (12%) had experienced WB, but not cyber-bullying, and only three (<1%) had been cyber-bullied, but did not meet the criterion for WB.

When asked to self-identify as having been bullied (either CB or WB), 138 (16.79%) reported yes, rarely or now and then, and 14 (1.7%) reported that they had been bullied several times a week or almost daily. Of the 152 who self-identified as having been bullied rarely or more often, 47 (31%) said the perpetrator was a supervisor, employer or manager; 74 (48%) said a peer; 27 (17%) said a subordinate; and 26 (17%) said a client. Participants could endorse more than one response.

Looking at the two control variables of gender and role, compared to men, women reported worse physical health, more strain, destructive leadership, team conflict and WB (Table 1). There were no significant gender differences for self-reported performance, absenteeism, ethical leadership, POS, effectiveness of organisational strategies or CB. Compared to managers, non-managers reported worse physical health, more strain, less POS, less effective organisational strategies and less CB, but there were no differences in performance, absenteeism, leadership, team conflict or WB.

Regression analyses were conducted to investigate the personal and organisational factors anticipated to predict both WB and CB at Times 1 and 2.

There was mixed evidence for the role of personal resources in relation to WB (Table 2).

Job performance and absenteeism were unrelated to WB (Hypotheses 1a(i) and 1d(i)). However, those with worse physical health and higher strain at Time 1 experienced more bullying at Time 2 (Hypotheses 1b(i) and 1c(i)). There was stronger support for the importance of organisational factors in WB. While positive organisational resources, such as ethical leadership and POS, were not related to WB (Hypotheses 2a(i) and 3(i)), destructive leadership, more team conflict and less effective organisational strategies at Time 1 were associated with higher levels of Time 2 WB (Hypotheses 2b(i), 4(i) and 5(i)).

In relation to CB, role was a significant predictor, but not gender: managerial employees experienced more CB behaviours. Poor physical health was associated with more CB (Hypothesis 1b(ii)), but strain (Hypothesis 1c(ii)), performance (Hypothesis 1a(ii)) and absenteeism (Hypothesis 1d(ii)) were not. Ethical and destructive leadership (Hypotheses 2a(ii) and 2b(ii)), and team conflict (Hypothesis 5(ii)) were unrelated to CB. However, higher levels of POS and more effective organisational responses were related to lower levels of CB (Hypothesis 3(ii) and 4(ii), respectively).

## 4. Discussion

The findings of the present study provided little support for the argument that personal and role resources (seniority, performance) would be related to fewer experiences of bullying. Employees with worse health were likely to experience more bullying, but this was not related to self-reported poorer work performance or absenteeism; those in worse health may be bullied for other reasons, such as perceived vulnerability, and as has been well established, ongoing bullying is highly damaging to both physical and mental health. Rather than identifying personal resources as fostering bullying, the study reinforced the importance of organisational factors.

Destructive leadership and high levels of team conflict were related to WB, but not CB. That the relationship is only with WB and not CB is somewhat surprising. While poor leadership and teamwork are known to be related to increased bullying, it is not clear why they appear to play a smaller role in CB. CB is private and generally invisible to others, making the role of leadership problematic [24], but workplace bullying can also be covert and difficult to manage. Those in managerial positions were more likely than non-managers to experience CB, but not WB. The perceived anonymity of CB may allow employees to bully “upward” without the risks of doing so face to face, but this requires further examination. Workplaces with clear policies and norms of behaviour, in which conflict is managed and where employees trust the effectiveness of systems, should be those in which bullying is less likely to flourish [12,53,56]. The findings support this argument, as perceptions that organisational strategies were effective was related to less WB and CB. Reduced CB was also related to perceptions that the organisation was more supportive; a supportive organisational climate may create fewer reasons for employees to bully each other, or it may provide reassurance that such behaviour will be addressed if it does occur.

Rates of CB were much lower than those of WB, and very few participants reported experiencing CB without also experiencing WB. This is in line with other research, which has found that CB may not create “new” targets, but becomes an additional means by which bullies can reach their targets [27,39]. Whether being targeted in multiple ways creates greater impacts on targets is an issue worth exploring; the small numbers of those experiencing CB in this study did not allow this issue to be examined.

### 4.1. Limitations

Some limitations of the present study need to be considered. First is the use of self-report data. Employees with worse psychological health may both perceive their work situation in a negative way and report more experiences of bullying, and those who are bullied may see their work environment more negatively as a result [5]. However, research has consistently shown that both non-bullied and bullied participants, including witnesses, report poor work environments in workplaces where bullying takes place [8,53], which casts doubt on the idea that targets of bullying are particularly biased in the ways in which they see their workplaces.

A second concern is with the use of multiple-item lists of negative acts. These measures cannot capture all possible negative behaviours, and no data are available on behaviours that are not included. A related concern is that the measures focus on the frequency of behaviours, but not the severity of the different acts; some negative acts may have more impact than others [82]. This is an issue worth exploring. It is also worth noting that the measurement of CB is still developing; the measure used in the present study is new and requires further assessment.

### 4.2. Directions for Future Research

Bullying and cyber-bullying deserve further study. In particular, there is a need to examine whether the prevalence and types of negative behaviour change over time, as well as the short-term and long-term impacts within organisations, using longer time frames than in the present study. The interrelationships between WB and CB also require further examination. It is as yet unclear whether CB is more problematic across organisational boundaries or within organisations, given the reach and scope that online technologies provide. In-depth examination of WB and CB in the context of one organization’s policies and practices would also provide opportunities to examine how approaches to managing negative behaviours are enacted and the impacts that they have. Further analysis could use modelling approaches to explore the reciprocal relationships between the antecedents and consequences of WB and CB.

## 5. Conclusions

Recommendations for addressing WB tend to revolve around the development of organisational solutions, such as policy, procedures, guidelines, systems, training and “cultures of respect” [11,30,65], but bullying remains a problem in many workplaces. Targets are often unwilling to report bullying and rarely feel that doing so would be beneficial; policies are often poorly implemented [11], and HR practitioners are often not prepared to support employees when managers are accused of bullying [83].

Similar problems are likely to arise in relation to CB. While our data show that perceived effectiveness of organisational strategies was related to less bullying, few organisations have established codes of cyber conduct or formal or informal means of controlling online behaviour beyond policies about appropriate and inappropriate Internet use [3,84]. Policies need to be implemented and monitored, and monitoring of employees’ online activities raises concerns about privacy [3]. Network providers can monitor and manage abusive communications, but “censoring” of online activity is often resented by all participants, not just bullies [85].

Some suggested solutions are aimed at targets, such as raising awareness about the need for caution in online interactions [25], building resilience [11] or changing the use of technologies. Targets are unwilling to report that they are experiencing cyber-bullying [33,86] and are understandably reluctant to abandon the technology that is being used to bully them. Less drastic steps, such as ignoring or blocking unwanted contacts, changing passwords or phone numbers, limiting online work activities to working hours and not adding unknown people to contacts lists may have some effect [25]; but, these do not address the root causes, and cyber-bullies may find other ways to reach their targets or choose new targets.

One distinctive feature of CB is that online activity leaves a relatively permanent trail. Even anonymous communications may be traceable, provided there is policy, skill and willingness to engage with the issue [86]. Organisations may be able to identify CB and take appropriate steps. However, this requires effective policy, which defines bullying (face to face or online), clarifies standards of acceptable and unacceptable behaviours, is implemented, communicated and supported, offers support for affected employees and considers privacy issues, confidentiality and the implications of ongoing technological changes [24]. To date, however, there is evidence that organisations are often ineffective in dealing with allegations of both WB and CB [10,33]. Clearly, there is room for improvement in organisational practice to manage this costly workplace problem.

## Figures and Tables

**Table 1:** Bivariate correlations between Time 1 variables and Time 2 workplace bullying and cyber-bullying. POS, perceived organisational support.

Variable Name	Mean	SD	1	2	3	4	5	6	7	8	9	10	11	12
1. Gender	-	-												
2. Role	-	-	0.16 **											
3. Performance	7.92	1.35	0.02	−0.03										
4. Physical health	1.63	0.50	0.19 **	0.070 *	−0.09 *									
5. Strain	2.26	0.516	0.09 *	0.10 **	−0.28 **	0.43 **								
6. Absenteeism	0.22	0.58	0.02	0.07	−0.07 *	0.12 **	0.07							
7. Ethical leadership	3.58	0.87	−0.04	−0.07	0.18 **	−0.23 **	−0.43 **	−0.13 **						
8. Destructive Leadership	1.91	0.81	0.09 *	0.01	−0.16 **	0.23 **	0.42 **	0.11 **	−0.75 **					
9. POS	5.03	1.59	−0.05	−0.17 **	0.20 **	−0.27 **	−0.51 **	−0.10 **	0.69 **	−0.62 **				
10. Team conflict	2.25	0.84	0.13 **	0.01	−0.08 *	0.28 **	0.33 **	0.04	−0.39 **	0.48 **	−0.42 **			
11. Effectiveness of org. strategies	2.96	0.66	−0.07	−0.12 **	0.17 **	−0.17 **	−0.44 **	−0.13 **	0.46 **	−0.43 **	0.52 **	−0.39 **		
12. Workplace bullying T2	1.36	0.47	0.07 *	−0.05	−0.10 **	0.34 **	0.40 **	0.09 **	−0.41 **	0.47 **	−0.41 **	0.47 **	−0.38 **	
13. Cyber-bullying T2	1.10	0.25	−0.00	−0.13 **	−0.01	0.16 **	0.21 **	0.07 *	−0.18 **	0.20 **	−0.24 **	0.19 **	−0.23 **	0.54 **

Notes: * *p* < 0.05; ** *p* < 0.01.

**Table 2:** Regression tests of hypotheses.

Variable Name	Workplace Bullying			Cyber-Bullying		
	B	SE(B)	Beta	B	SE(B)	Beta
Gender	−0.03	0.03	−0.03	−0.04	0.02	−0.08
Role	0.04	0.03	0.04	0.05	0.02	0.10 *
Performance	0.00	0.01	0.00	0.01	0.01	0.07
Absenteeism	0.05	0.03	0.07 *	0.03	0.02	0.07
Physical health	0.13	0.04	0.15 ***	0.05	0.03	0.10 *
Strain	0.10	0.04	0.11 *	0.04	0.03	0.07
Ethical leadership	0.01	0.03	0.02	0.03	0.02	0.08
Destructive leadership	0.13	0.03	0.22 ***	−0.00	0.02	−0.01
POS	−0.02	0.02	−0.08	−0.04	0.01	−0.21 **
Team Conflict	0.11	0.02	0.20 ***	0.00	0.02	0.00
Effectiveness of org. responses	−0.08	0.03	−0.11 **	−0.07	0.02	−0.16 **
*F*	*30.64* ***			*7.11* ***		
*R^2^*	0*.38*			0*.13*		
*R^2^ adj.*	0*.37*			0*.11*		

Notes: * *p* < 0.05; ** *p* < 0.01; *** *p* < 0.001.
